# Self-locomotion and spatial language and spatial cognition: insights from typical and atypical development

**DOI:** 10.3389/fpsyg.2014.00521

**Published:** 2014-06-02

**Authors:** Ora Oudgenoeg-Paz, James Rivière

**Affiliations:** ^1^Department of Special Education, Centre for Cognitive and Motor Development, Utrecht UniversityUtrecht, Netherlands; ^2^Laboratoire Psychologie et Neurosciences de la Cognition, Université de RouenMont Saint Aignan, France

**Keywords:** spatial cognition, spatial language, spatial exploration, motor impairment, spinal muscular atrophy, self-locomotion

## Abstract

Various studies have shown that occurrence of locomotion in infancy is correlated with the development of spatial cognitive competencies. Recent evidence suggests that locomotor experience might also be important for the development of spatial language. Together these findings suggest that locomotor experience might play a crucial role in the development of linguistic-cognitive spatial skills. However, some studies indicate that, despite their total deprivation of locomotor experience, young children with spinal muscular atrophy (SMA) have the capacity to acquire and use rich spatial representations including good spatial language. Nonetheless, we have to be cautious about what the striking performances displayed by SMA children can reveal on the link between motor and spatial development, as the dynamics of brain development in atypically developing children are different from typically developing children.

## INTRODUCTION

Developmentalists widely agree that many factors potentially influence spatial cognitive-linguistic development in infancy. The embodied cognition approach stresses motor development as one of the main factors to be considered in this aspect (Smith and Gasser, 2005; Rakison and Woodward, 2008; Hockema and Smith, 2009). According to this view, spatial language and spatial cognition are grounded in sensorimotor interaction with the environment. Children perceive the spatial structure of their environment (through vision, hearing etc.), *and* children also act on the world and change the spatial structure of their environment. These ongoing perception-action cycles form the basis on which spatial language and spatial cognition emerge (Gibson and Pick, 2000; Smith and Gasser, 2005). Attainment of motor milestones plays an important role in this process, as it changes the way children interact with their environment. Attainment of self-locomotion is thought to be especially important for the development of spatial (linguistic and cognitive) skills. Once infants can engage in self-locomotion, they focus their attention on information needed to guide their locomotion (e.g., information about the spatial arrangement). Moreover, children can manipulate the environmental spatial arrangement more easily when engaged in self-locomotion (Gibson, 1988; Campos et al., 2000).

In the current paper we first present a number of studies investigating the relationship between self-locomotion and spatial cognition and spatial language in typically developing children. We review evidence from both cross-sectional and longitudinal studies and end this section with a short discussion of a few relevant questions. Second, we present the results of several studies in children with spinal muscular atrophy (SMA), a hereditary neuromuscular disease which results in severe motor impairments. The correlations between self-locomotion and the development of spatial linguistic-cognitive competencies could lead to the suggestion that locomotor impairment might be a risk factor impeding the development of these competencies. However, despite their total deprivation of locomotor experience, children with SMA have the capacity to acquire and use rich spatial representations. We suggest a few hypotheses explaining the spatial capacities of these children and discuss the meaning of these findings for the embodied cognition perspective.

## SELF-LOCOMOTION, SPATIAL LANGUAGE, AND SPATIAL COGNITION IN TYPICALLY DEVELOPING CHILDREN

Studies with typically developing children have consistently demonstrated relations between the attainment of self-locomotion and spatial skills. Many studies have focused on children’s spatial search abilities and specifically on the A-not-B task. In this task, originally used by Piaget (1952), children view a toy being hidden in one of two identical locations (the A location). After a delay children are allowed to search for the toy. After having found the toy at location A on a few consecutive trials, the toy is hidden at the B location (Diamond et al., 1997). Young infants usually continue searching in the A location and thus make the A-not-B error. Around 12 months infants stop making this error and this change appears to be tightly related to the acquisition of self-locomotion. Kermoian and Campos (1988) have shown that 8.5-months-old infants who could crawl on hands and knees, or had walked using a baby-walker, were better at this task than infants of the same age who had not had any experience with self-locomotion. In a similar study, Bai and Bertenthal (1992) showed with infants aged 7.5 months that the length of experience with self-locomotion (either hands and knees crawl or using a baby-walker) positively predicted performance on the task. Infants with less than four weeks locomotion experience did not perform better than prelocomotor infants. These findings suggest that amount of experience with self-locomotion is the important factor.

Clearfield (2004) has shown that infants (aged 8, 11, and 14 months) with 6 weeks or more experience with self-locomotion (crawling or walking) were better at finding toys in a large space than novice crawlers and walkers. There appears to be no transfer of effects between crawling and walking as novice walkers performed as poorly as novice crawlers. However, the design of this study does not enable disentangling the effects from a possible confound with age. Work by Berger (2010) showed similar results. In this study, 13-months-old crawling and walking infants performed an adapted version of the A-not-B task where they had to reach their caregiver by following either (1) a direct path (2) an indirect path (3) a direct path through a tunnel. Novice crawlers and walkers performed worse than expert crawlers and walkers on these tasks. Taken together these studies suggest that experience with hands and knees crawl and walking predicts success in spatial search tasks.

Mental rotation skills have also been related to the attainment of self-locomotion. Schwarzer et al. (2013) have shown that 9-months-old crawling infants looked significantly longer at the mirror image of a previously seen object than at the image of the original object. This suggests they could mentally rotate the object and could therefore see that the mirror image is a new object. Same age non-crawling infants showed no difference in looking time. Frick and Möhring (2013) showed, using a similar task that 8 and 10-months-olds that could walk with assistance were better at this task than children who could not yet walk with assistance even after controlling for age. Without controlling for age, an earlier age of crawling also predicted better performance on this task.

Within the spatial skills, spatial language is the skill of communicating about spatial information (Landau and Jackendoff, 1993). Empirical studies have provided compelling support for a link between spatial cognition and spatial language (see for example: Landau and Hoffman, 2005; Wallentin et al., 2005). Therefore it seems logical that spatial language will also be related to self-locomotion. However, evidence pertaining to this relation in typically developing children is scarce. A recent longitudinal study from our lab showed that Dutch children who started walking at an earlier age had better spatial language (measured as locative prepositions and verbs containing movement in a specific direction) at age 32 months (Oudgenoeg-Paz et al., 2013).

While this study demonstrated longitudinal relations between self-locomotion and spatial language, most of the studies demonstrating a link between self-locomotion and spatial cognition were cross-sectional. Only a few studies examined these relations longitudinally. Murray et al. (2006) have shown that earlier attainment of self-locomotion predicted better visuospatial memory far into adulthood. In contrast, work from our lab showed that the age of attainment of self-locomotion milestones did not predict spatial memory at ages 4 and 6 years. Engagement in spatial exploration (e.g., moving around a lot, playing with blocks and nesting cups) did significantly predict better spatial memory at ages 4 and 6 years. Age of self-locomotion, in turn, predicted more engagement in spatial exploration. The lack of relations between self-locomotion and spatial memory, might therefore suggest that children who initially lag behind on motor development do catch up, but at the long term it is exploration (which is initially enabled by motor development) which is important for future spatial skills (Oudgenoeg-Paz et al., 2014).

Thus, exploration behavior (the way children interact with their environment) might be one of the mechanisms underlying the relation between self-locomotion and spatial language and cognition. Support for this hypothesis comes from the study previously discussed (Oudgenoeg-Paz et al., 2013) where we found that children who started walking at an earlier age, moved around more during exploration also at age 20 months (an age in which all children could walk). These children also had better spatial language at age 32 months. Exploration through self-locomotion partially mediated the effect of age of walking on spatial language.

It is important to note that while the studies reviewed here support the link between self-locomotion and spatial skills such as spatial language and spatial memory, evidence regarding other skills such as spatial coding is less compelling (see for example: Tyler and McKenzie, 1990; Bell and Fox, 1997).

### OUTSTANDING QUESTIONS

Taken together, these results suggest that exploration is one possible candidate for a mechanism underlying the relations between the attainment of self-locomotion and spatial linguistic-cognitive skills. Exploration, however, is probably not the only mechanism. Other factors, such as social stimulation and attentional abilities (see for example: Gogate and Hollich, 2010; Karasik et al., 2011; Walle and Campos, 2014) are also possible candidates. More work is needed to gain insights into the role of self-locomotion in the development of spatial language and into different mechanisms underlying these relations. Another issue to be addressed is the question whether self-locomotion is a sufficient or necessary condition for the development of spatial linguistic-cognitive skills. The presence of underlying mechanisms, such as exploration implies that self-locomotion is not sufficient. Factors such as materials in the child’s environment and the child’s drive to explore probably also play a role. To answer the question whether self-locomotion is necessary for the development of spatial language and spatial cognition we turn to evidence from children with motor impairments.

## SPATIAL LANGUAGE AND SPATIAL COGNITION IN YOUNG CHILDREN WITH SPINAL MUSCULAR ATROPHY

Spinal muscular atrophy is a hereditary neuromuscular disease characterized by severe progressive muscular weakness due to a degeneration of the anterior horn cells of the spinal cord. This rare genetic disorder (incidence = 1 out of 6,000 newborns) is caused by a microdeletion on chromosome 5q13 (Melki et al., 1994). Clinical manifestations are severe muscular weakness; proximal limb muscles are more affected than distal muscles, and lower limb muscles more than upper muscles. The clinical evolution is characterized by degeneration which varies in rapidity depending on the type. SMA is divided into types 1, 2, and 3; classification is based on the age of onset, developmental milestones, and life span.

Type 2 - SMA is the intermediate form, with onset between 6 and 18 months of age (Munsat, 1991). Children with intermediate SMA are unable to crawl and walk. Most of these patients could sit within the normal age range (up to 9 months), the remainder learned to do so between 10 and 30 months (Bertini et al., 2005). Patients with type 2 - SMA who can stand up have a better prognosis than those who cannot. Indeed, those who can stand up generally do not have breathing impairment and rarely have distal upper limb weakness in the first 5 years of life (Zerres et al., 1997).

In our laboratory, three experiments were conducted in order to test spatial linguistic-cognitive skills in young children with type 2 - SMA. These children had never crawled or walked (at any level) and they had never driven a motorized wheelchair. In the first study (Rivière and Lécuyer, 2002), 12 type-2 SMA children, from 21 to 36 months old (mean age = 30 months), were compared with chronological age-matched controls with respect to their spatial search skills in a memory-for-location task. In this task, several cups, with one hiding a small object, were placed on a rotating tray which was turned 180° before a search was permitted. There was no difference in performance between SMA children and the healthy control group. In the second study (Rivière and Lécuyer, 2003), 14 type-2 SMA children, from 20 to 36 months old (mean age = 29 months) and 14 chronologically age-matched controls were presented with a 3-location search task involving the invisible displacements of an object. Results show that the performance of the SMA group was significantly superior to that of the healthy control group. The third study (Rivière et al., 2009) examined the comprehension and production of linguistic markers of spatial relations in two groups of French-speaking children: a group of 12 type-2 SMA children aged from 24 to 37 months (mean age = 33 months) and a chronologically age-matched control group. Results showed no difference between healthy and SMA children in the comprehension task. In the production task, SMA children were more successful than the control group. The SMA children showed particularly significantly more advanced performance, with the prepositions “in front of” and “behind,” which are among the most difficult prepositions during normal language acquisition. Furthermore, this advantage appears particularly in the production task, which is more difficult than the comprehension task. The performance of SMA children suggests that, despite their total deprivation of locomotor experience, they have the capacity to acquire and use rich spatial representations that are embodied in the semantics of natural languages.

Taken together, these results indicate that young children with SMA excel in spatial language and spatial cognition. Different hypotheses have been proposed to explain these striking findings.

### SOCIAL STIMULATION HYPOTHESIS

For type-2 SMA children having no experience with locomotion, language is a particularly crucial tool during their cognitive development. Because of their severe motor impairment, type-2 SMA children cannot actively transform their physical environment. However, they are skilled in manipulating their caregivers in order to get them to act on the environment. In this respect, language enables these children to transform their physical environment, despite the fact that it is unreachable for them. Consequently, the results concerning SMA children may not be surprising. Since these children are motorically impaired, they rely more on language than healthy children in order to get other people around them to perform actions for them. It might be that this way these children obtain the same information typically developing children obtain through active exploration.

It should be also noted that language can play a role in structuring spatial cognition (cf. Hermer-Vazquez et al., 2001). Recent cross-linguistic work has established that frames of reference (i.e., coordinate systems used to compute and specify the location of objects with respect to other objects) vary across languages and cultures (cf. Majid et al., 2004). The hypothesis according to which the acquisition and use of language favors the development of spatial representations could explain the cognitive profile of children with SMA. Indeed, these patients exhibit both rich knowledge of the linguistic markers of spatial relationships, and high scores in spatial search tasks. One can speculate that, by providing both regularities in the child’s environment and linguistic inputs, social stimulations foster spatial cognition. As a result, a social stimulation hypothesis can be put forth to account for the striking cognitive performances in young SMA children. Evidence from studies with typically developing children suggests that they too profit from social interaction to stress regularities in the learning of language and spatial concepts (cf. Pruden et al., 2011; Pereira et al., 2014). However, one can also speculate that differences in activation of lower-level mechanisms could result in distinct higher-level cognitive skills. Thus, in SMA children, increased attentional abilities could be a sufficient explanation for the excellent cognitive performances observed.

### IMPROVED ATTENTION HYPOTHESIS

According to Rivière and Lécuyer (2003), the excellent levels of performance displayed by young SMA children in spatial cognition may be attained through an increased importance of visual attention to the environment. Indeed, clinicians have noted the keen interest of these children in their surroundings, their observational abilities and their mental acuity (cf. von Gontard et al., 2002). The major role of visual attentional capacities in memory for spatial locations in typically developing children was underlined by several authors (e.g., Horobin and Acredolo, 1986; Foreman et al., 1990, 1994). These findings led Rivière and Lécuyer (2003) to consider the possibility that SMA children allocate more attentional resources than typically developing children when completing spatial search tasks. This suggestion is compatible with experimental data. Thus, Rivière and Lécuyer (2003) reported that in the 3-location search task where children with SMA outperformed than the healthy controls, SMA children were slower in starting their reaching movement in the first but not in the second trial. Given the difference in reaction time between the trials, the difference between SMA children and the control group cannot result only from differences in motor capacities, and thus cannot be explained in purely mechanical terms. These findings support the view that SMA children allocate more attentional resources than healthy controls for the processing of the spatial search task.

### OUTSTANDING QUESTIONS

Further research is needed to address the question of whether the striking cognitive performances in young SMA children are at least in part based on different processing strategies than those used by healthy children. Four different types of studies would be helpful in testing the viability of this hypothesis:

(1) Extensive task analyses that evaluate the qualitative differences in cognitive abilities of SMA children;
(2) Longitudinal prospective studies beginning in early or late infancy that asses linguistic and cognitive skills in SMA children in order to acquire a full understanding of their developmental course;
(3) Investigations of lower-level mechanisms, targeting factors thought to contribute to genesis of exceptional performances in SMA children, especially attentional abilities;
(4) Neuroimaging studies investigating the brain activity of young children with SMA in order to know whether the apoptosis of spinal motor neurons in SMA may stimulate a cortical re-mapping.

## CONCLUSION

Spatial language and spatial cognition are complex systems encompassing different types of processes. The correlations between self-locomotion in infancy and the development of spatial language and spatial cognition in typically developing children could be attributed to a number of underlying mechanisms such as exploration behavior, social stimulation or attentional abilities. The latter mechanisms could explain the excellent performances of SMA children on linguistic-cognitive spatial tasks. It is clear that while the attainment of self-locomotion is linked to the development of spatial skills in typically developing children it is neither a sufficient nor a necessary condition. In the pathways leading to the development of these skills, both typically and atypically developing children may follow alternative paths.

However, we have to be cautious about what the striking performances displayed by SMA children can reveal on the relation between motor and spatial development, as atypical development cannot always provide us with a window to typical development. The dynamics of brain development in atypically developing children are different from typically developing children on many levels. This in turn may imply that the mechanisms underlying development in these children are not always the same as in typically developing children (cf. Karmiloff-Smith, 1998).

## Conflict of Interest Statement

The authors declare that the research was conducted in the absence of any commercial or financial relationships that could be construed as a potential conflict of interest.
